# Recurrent SKIL-activating rearrangements in ETS-negative prostate cancer

**DOI:** 10.18632/oncotarget.3359

**Published:** 2015-01-31

**Authors:** Matti Annala, Kati Kivinummi, Joonas Tuominen, Serdar Karakurt, Kirsi Granberg, Leena Latonen, Antti Ylipää, Liisa Sjöblom, Pekka Ruusuvuori, Outi Saramäki, Kirsi M. Kaukoniemi, Olli Yli-Harja, Robert L. Vessella, Teuvo L.J Tammela, Wei Zhang, Tapio Visakorpi, Matti Nykter

**Affiliations:** ^1^ Institute of Biosciences and Medical Technology - BioMediTech, University of Tampere, Tampere, Finland; ^2^ Institute of Biosciences and Medical Technology - BioMediTech, Tampere University of Technology, Tampere, Finland; ^3^ Fimlab Laboratories, Tampere University Hospital, Tampere, Finland; ^4^ Department of Urology, University of Washington, Seattle, WA, USA; ^5^ Department of Urology, Tampere University Hospital and Medical School, University of Tampere, Tampere, Finland; ^6^ Department of Pathology, University of Texas M.D. Anderson Cancer Center, Houston, TX, USA

**Keywords:** prostate cancer, sequencing, fusion gene, SKIL

## Abstract

Prostate cancer is the third most common cause of male cancer death in developed countries, and one of the most comprehensively characterized human cancers. Roughly 60% of prostate cancers harbor gene fusions that juxtapose ETS-family transcription factors with androgen regulated promoters. A second subtype, characterized by *SPINK1* overexpression, accounts for 15% of prostate cancers. Here we report the discovery of a new prostate cancer subtype characterized by rearrangements juxtaposing the SMAD inhibitor *SKIL* with androgen regulated promoters, leading to increased *SKIL* expression. *SKIL* fusions were found in 6 of 540 (1.1%) prostate cancers and 1 of 27 (3.7%) cell lines and xenografts. 6 of 7 *SKIL*-positive cancers were negative for *ETS* overexpression, suggesting mutual exclusivity with *ETS* fusions. *SKIL* knockdown led to growth arrest in PC-3 and LNCaP cell line models of prostate cancer, and its overexpression led to increased invasiveness in RWPE-1 cells. The role of *SKIL* as a prostate cancer oncogene lends support to recent studies on the role of TGF-β signaling as a rate-limiting step in prostate cancer progression. Our findings highlight *SKIL* as an oncogene and potential therapeutic target in 1-2% of prostate cancers, amounting to an estimated 10,000 cancer diagnoses per year worldwide.

## INTRODUCTION

Prostate cancer is diagnosed in over 900,000 men worldwide every year, making it the second most common cancer among men [1]. The standard-of-care for localized prostate cancer is radical prostatectomy or radiation therapy, whereas advanced tumors are treated with systemic therapies that inhibit androgen signaling [2]. More specific drug targets and driver mutations have been sought through extensive genomic characterization efforts [3,4]. We now know that genomic rearrangement plays a major role in the onset of prostate cancer, with 60% of tumors harboring chromosomal rearrangements that juxtapose androgen regulated promoters with the ETS family transcription factors *ERG, ETV1, ETV4* or *FLI1* [5,6]. However, the products of these fusion genes have proven difficult to target with small molecule inhibitors. More recently, overexpression of the trypsin inhibitor SPINK1 was found to define a second prostate cancer subtype mutually exclusive with ETS overexpression [7]. Monoclonal SPINK1 antibodies have shown efficacy in preclinical models [8], suggesting that SPINK1 inhibition may prove a beneficial treatment strategy in the 15% of prostate cancers positive for SPINK1 overexpression. Recent studies have identified other alterations mutually exclusive with ETS fusions, including mutations in the *SPOP* gene [4], and deletions of the chromatin remodeling gene *CHD1* [9]. Despite these discoveries, a significant fraction of prostate cancers do not harbor any of the above alterations.

In addition to ETS fusions and associated events, genomic characterization studies have identified non-synonymous mutations in *TP53*, *MED12*, and *PTEN* [3,4,10], and gross deletions of the tumor suppressor genes *PTEN*, *RB1* and *TP53* [3,4,10]. More recently, a number of studies have highlighted the role of attenuated TGF-β signaling in prostate cancer progression [11-13]. SMAD4, a critical component of the TGF-β signaling cascade, is inactivated in a subset of advanced prostate cancers through promoter hypermethylation [14] or somatic mutation [10], and its expression is reduced in metastatic prostate cancer [11]. Mouse studies have shown that TGF-β signaling inhibits progression of *PTEN*-null tumors, and that *SMAD4* deletion can overcome this inhibition [11]. In pancreatic adenocarcinoma, biallelic inactivation of *SMAD4* is observed in 50% of tumors [15].

In this study, we performed transcriptome and low-coverage whole genome sequencing on 28 untreated and 13 castration resistant prostate cancers, and identified a new prostate cancer subtype characterized by activating rearrangements of the SMAD inhibitor SKIL.

## RESULTS

### Sample acquisition and sequencing

Fresh-frozen tissue from 12 benign prostatic hyperplasias (BPH), 28 untreated prostate cancers (PC), and 13 castration resistant prostate cancers (CRPC) was acquired from the Tampere University Hospital (Tampere, Finland). All samples contained a minimum of 70% cancerous or hyperplastic epithelial cells. PC samples were obtained by radical prostatectomy and locally recurrent CRPCs by transurethral resection of the prostate (Supplementary Table 1). Mean age at diagnosis was 60.8 years (range: 47.4-71.8) and mean PSA at diagnosis was 10.8 ng/ml (range: 3.5-48.1). Libraries were prepared for paired-end analysis on the Illumina HiSeq 2000. On average, we obtained 150 million paired end reads per sample from the low coverage whole genome sequencing, and 110 million paired end reads from the whole transcriptome sequencing (Supplementary Table 2).

### Discovery of recurrent SKIL-activating rearrangements

**Figure 1:**
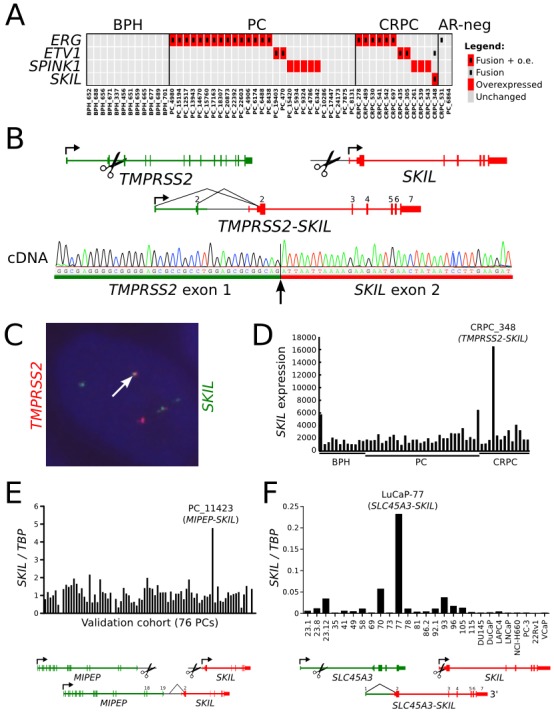
Recurrent *SKIL*-activating rearrangements in prostate cancer (A) Matrix showing mutually exclusive overexpression of *ERG*, *ETV1*, *ETV4*, *SPINK1*, and *SKIL* in a transcriptome sequencing cohort of 41 prostate cancers. Red rectangles indicate overexpression, and black inner rectangles indicate fusion events. (B) Structure of the *TMPRSS2-SKIL* fusion gene identified in sample CRPC_348. Black lines indicate exon-exon junctions with transcriptome sequencing evidence. Fusion transcript was validated with Sanger sequencing from cDNA. (C) Fluorescence in situ hybridization validates the fusion at genomic level. One example of a fusion positive cell is shown. (D) *SKIL* expression in the transcriptome sequencing cohort of 41 prostate cancers and 12 BPHs. *SKIL* is strongly overexpressed in the TMPRSS2-SKIL positive sample. (E) *SKIL* expression was measured using qRT-PCR in a validation cohort of 76 prostatectomy samples. Sample PC_11423 exhibited SKIL overexpression and was found to contain a *MIPEP-SKIL* rearrangement by transcriptome sequencing. (F) *SKIL* expression was measured using qRT-PCR in LuCaP xenografts and cell line models of prostate cancer. Xenograft LuCaP-77 was found to contain an *SLC45A3-SKIL* rearrangement by transcriptome sequencing.

**Figure 2:**
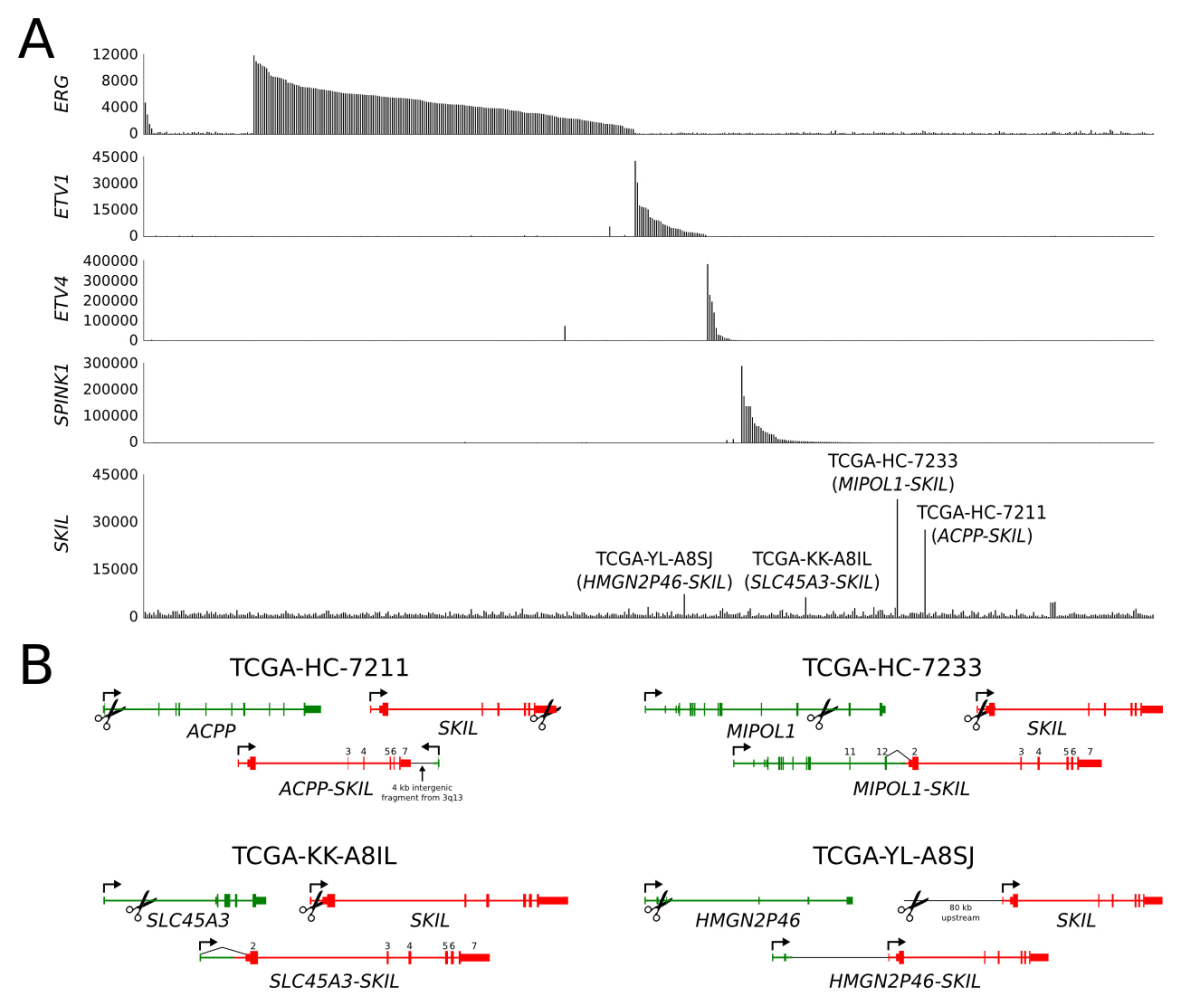
*SKIL*-activating rearrangements in the TCGA prostate adenocarcinoma sequencing cohort (A) Barplot showing expression of *ERG*, *ETV1*, *ETV4*, *SPINK1* and *SKIL* in TCGA samples. Four samples exhibited significant *SKIL* overexpression and were found to harbor *SKIL*-activating rearrangements. (B) Structures of the *ACPP-SKIL*, *SLC45A3-SKIL*, *MIPOL1-SKIL* and *HMGN2P46-SKIL* rearrangements. Black lines indicate exon-exon junctions with transcriptome sequencing evidence.

Fusion events involving an ETS family transcription factor or *SPINK1* overexpression were identified in 32 of 41 tumors based on transcriptome sequencing (Figure 1A, Supplementary Table 3). No SPOP mutations were identified in our cohort. We identified a novel *TMPRSS2-SKIL* fusion gene in one CRPC sample (Figure 1A-B, Supplementary Figure 1) and validated it using Sanger sequencing (Figure 1B) and fluorescence in situ hybridization (Figure 1C). The fusion merged the first three exons of *TMPRSS2* with full length *SKIL* and led to *SKIL* overexpression due to the androgen regulated *TMPRSS2* promoter (Figure 1D). *SKIL* encodes a SKI-like protein that inhibits TGF-β signaling by binding to and disrupting the heteromeric SMAD complex [16]. To search for more positive cases, we screened 76 additional tumors (Supplementary Table 1) and 22 LuCaP xenografts with qRT-PCR, and identified *SKIL* overexpression in one xenograft and one clinical sample. Transcriptome sequencing of these samples revealed a *SLC45A3-SKIL* fusion in LuCaP-77 and a *MIPEP-SKIL* fusion in the clinical sample, confirming *SKIL* as a recurrent 3′ fusion partner in prostate cancer (Figure 1E-F). Analysis of transcriptome sequencing data from the Cancer Genome Atlas (TCGA) prostate adenocarcinoma project revealed additional *SKIL*-activating rearrangements in 4 of 423 samples, with concomitant *SKIL* overexpression (Figure 2A-B). In the Taylor et al. dataset, two ETS-negative samples (PCA0015 and PCA0056) exhibited outlier overexpression of *SKIL*, but were excluded from further analysis due to lack of sequencing data [3]. Interestingly, all 5 SKIL-positive clinical samples with clinical information (two TCGA samples lacked clinical data) contained a Gleason grade 5 component or represented metastatic prostate cancer, suggesting that SKIL-activating alterations may associate with high-grade prostate cancer.

None of the seven *SKIL* rearrangements disrupted the protein coding sequence of *SKIL*, suggesting that full-length SKIL protein is necessary for oncogenic function. 6 of 7 *SKIL* rearrangements involved an androgen regulated promoter, indicating selection towards juxtapositions with highly active promoters. 6 of 7 fusion positive samples were negative for *ETS* overexpression, suggesting mutual exclusivity between *SKIL* and *ETS* rearrangements (p = 0.047, Fisher's exact test). Sample TCGA-YL-A8SJ overexpressed both *SKIL* and *ETV1* (Figure 2A), although we found no reads supporting an *ETV1* rearrangement in either the transcriptome or exome sequencing data for this sample.

In sample TCGA-HC-7211, the rearrangement between *ACPP* and *SKIL* had an unexpected structure, with the first exon and promoter of *ACPP* placed downstream of *SKIL* in antisense orientation (Figure 2B). Despite the non-canonical structure, the rearrangement led to strong overexpression of full-length *SKIL* (Figure 2A), possibly due to chromatin remodeling induced by the androgen regulated *ACPP* promoter. The antisense promoter provoked expression of a spliced antisense transcript composed of cryptic exons located in *SKIL* introns (Supplementary Figure 2). Expression of the antisense transcript did not appear to interfere with *SKIL* splicing, as sense transcripts were normally spliced.

### Level of nuclear SKIL protein is elevated in a fusion positive sample

**Figure 3:**
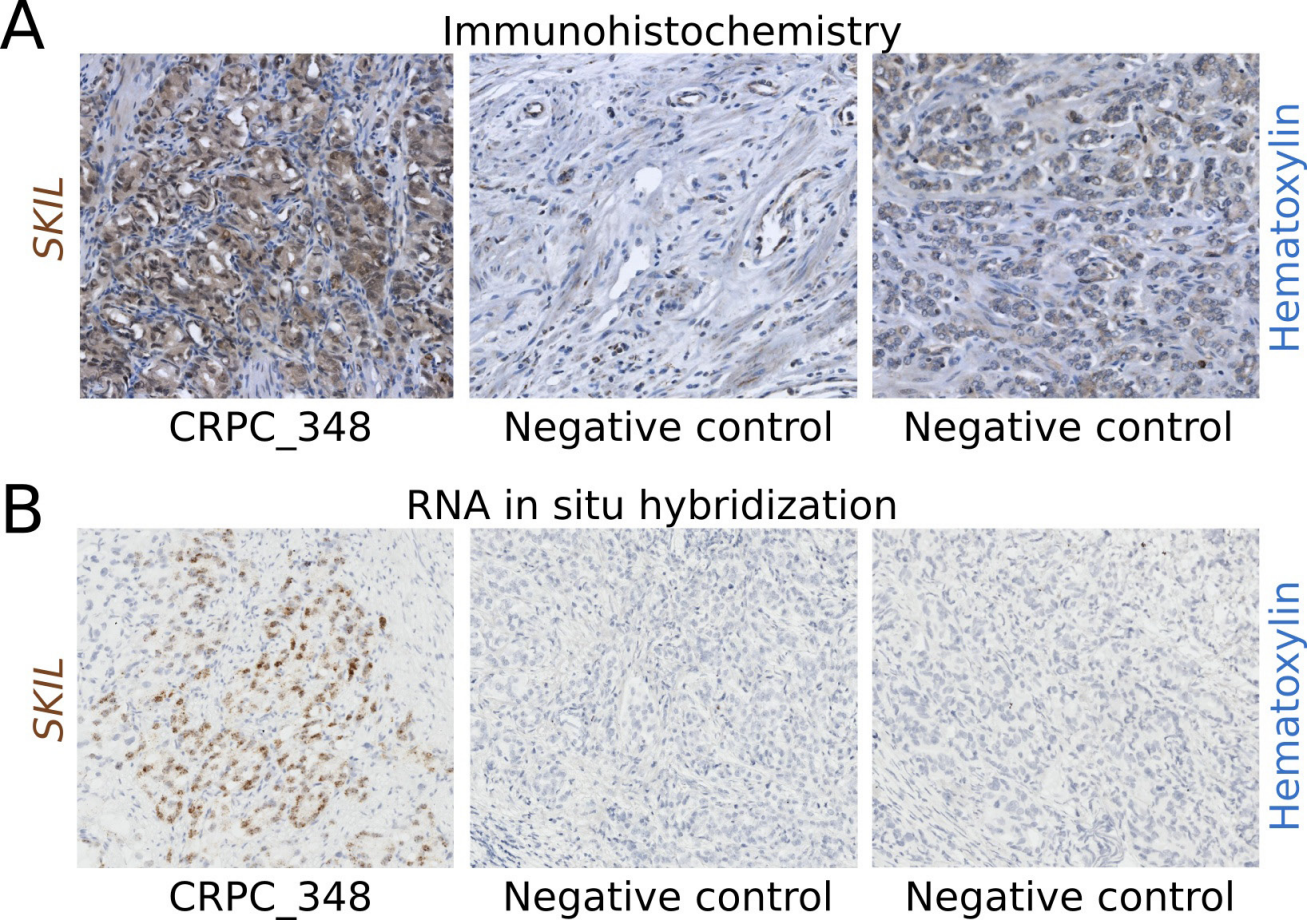
Immunohistochemistry and RNA in situ hybridization of SKIL in a SKIL-rearranged tumor (A) Anti-SKIL staining of paraffin-embedded sections from the *SKIL*-rearranged TURP sample CRPC_348 and two representative prostatectomies negative for *SKIL* rearrangement showing no staining or weak cytoplasmic staining. (B) RNA in situ hybridization of CRPC_348 and two representative prostatectomies negative for *SKIL* rearrangement, with probes targeting *SKIL* mRNA. Nuclei were stained with hematoxylin.

To determine whether fusion positive clinical samples also overexpressed SKIL at the protein level, we used a monoclonal antibody to perform immunohistochemistry on the fusion positive TURP sample CRPC_348 and 8 negative controls. The TURP sample exhibited strong nuclear and modest cytoplasmic staining for SKIL, while negative controls showed no staining or only weak cytoplasmic staining for SKIL (Figure 3A). We also showed the overexpression of *SKIL* at the RNA level in CRPC_348 using RNA in situ hybridization (Figure 3B).

### SKIL regulates proliferation and invasiveness of prostate cancer cells

**Figure 4:**
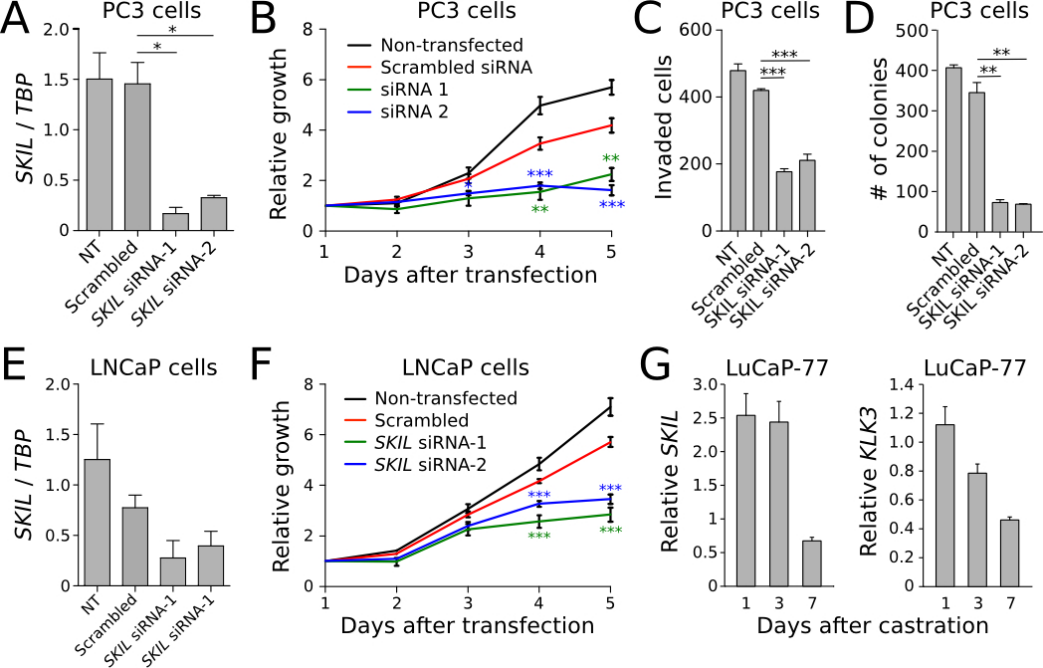
*In vitro* knockdown experiments on *SKIL* (A) *SKIL* expression was silenced in PC3 cells using two siRNAs, resulting in (B) reduced growth (n = 4), (C) invasion (n = 4) and (D) colony formation (n = 2). (E) SKIL expression was silenced in LNCaP cells using two siRNAs, resulting in (F) reduced growth (n = 4). (G) qRT-PCR time series of *SKIL* and *PSA* expression in castrate and non-castrate mice carrying LuCaP-77 xenografts (n = 2). Error bars, s.e.m. with first-order error propagation; *P<0.05; **P<0.01; ***P<0.001, unpaired two-tailed t-test.

To better understand the biological role of SKIL in prostate cancer cells, we knocked SKIL down in PC-3 cells using two different siRNA (Figure 4A), and observed reduced cell growth (Figure 4B), invasiveness (Figure 4C), and colony formation (Figure 4D) relative to scrambled siRNA. The effect on cell growth was replicated in LNCaP cells (Figure 4E-F). To show that *SKIL* expression is truly androgen dependent in cells where *SKIL* is fused with androgen regulated promoters, we extracted LuCaP-77 xenograft tissue (with an *SLC45A3-SKIL* fusion) from castrate and non-castrate mice 1, 3 and 7 days post-castration. We then used qRT-PCR to quantify *SKIL* and *KLK3* (PSA) expression at each timepoint. Expression values were normalized against TATA-box binding protein (*TBP*) and compared against non-castrate control mice. We observed a strong reduction in the expression of both *KLK3* and *SKIL* on day 7, indicating androgen regulated expression (Figure 4G).

**Figure 5:**
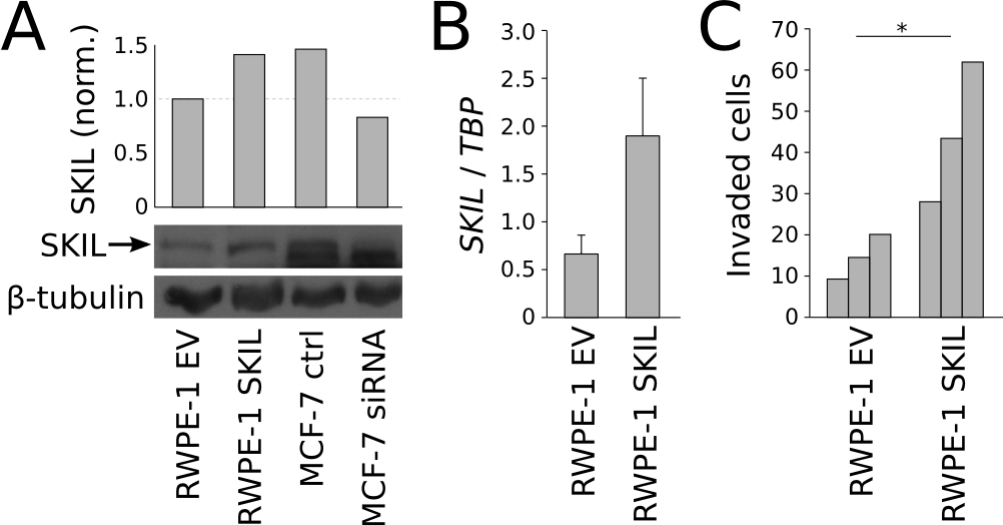
*In vitro* overexpression experiments on *SKIL* (A) Anti-SKIL western blot showing increased SKIL protein in SKIL-transfected RWPE-1 cells. MCF-7 cells transfected with SKIL or scrambled siRNA are used to validate the band. (B) qRT-PCR quantification of *SKIL* in RWPE-1 cells transfected with SKIL or empty vector. (C) Matrigen invasion assay on RWPE-1 cells transfected with SKIL or empty vector (n = 3). Error bars, s.e.m. with first-order error propagation; *P<0.05; **P<0.01; ***P<0.001, unpaired two-tailed t-test.

Next, we created a SKIL overexpression model by transfecting immortalized prostate epithelial cells (RWPE-1) with a pCI-Neo vector expressing hemagglutinin (HA) tagged SKIL [17]. Control RWPE-1 cells were transfected with an empty pCI-Neo vector (EV). In comparison to control cells, *SKIL*-transfected RWPE-1 cells exhibited higher expression of SKIL at both RNA and protein levels (Figure 5A-B), and greater invasive potential in a matrigel invasion assay (n = 3, p = 0.044, unpaired two-tailed t-test) (Figure 5C). SKIL overexpression had no effect on the growth of RWPE-1 cells (data not shown).

### Other genomic alterations in the sequencing cohort

A previous study has shown that combined deletion of *SMAD4* and *PTEN* in mouse prostates leads to aggressive prostate cancer with 100% penetrance [11]. Therefore, we set out to check whether *SKIL*-rearranged cancers harbored concomitant *PTEN* deletions. *PTEN* expression was not aberrantly low in any of the 7 SKIL-positive cases, and only 1 of 4 SKIL-positive cancers in the TCGA cohort showed evidence of *PTEN* deletion, suggesting that *SKIL* rearrangements do not require combined *PTEN* loss. Genes associated with *ERG* overexpression, such as *COL2A1*, *ALOX15*, *CRISP3, B3GNT6* and *TDRD1*, were not overexpressed in SKIL-rearranged tumors.

**Figure 6:**
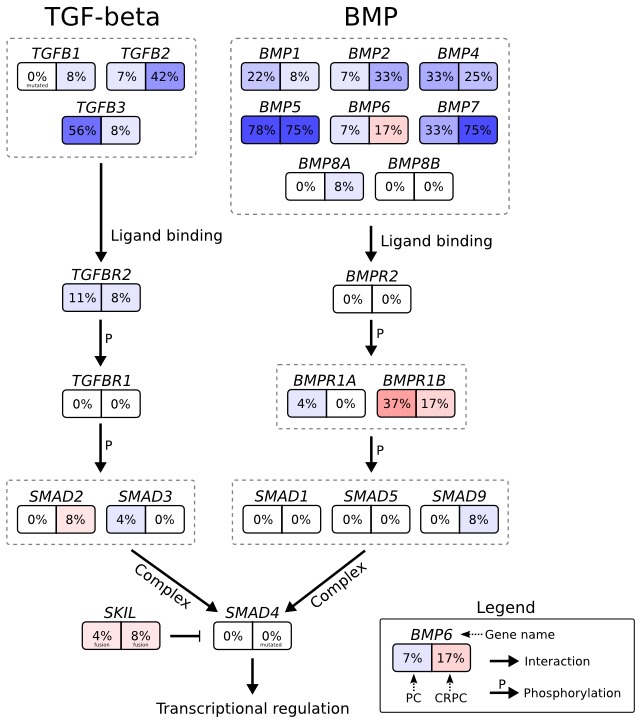
Genomic and transcriptomic changes in the context of the TGF-β signaling pathway Genes are shown as boxes with two halves: the left half shows the percentage of untreated prostate cancers with two-fold upregulation (red) or downregulation (blue) relative to BPH, and the right half shows the same for castration resistant prostate cancers. Arrows indicate interactions between proteins or genes, the interaction type is written next to the arrow.

Expression analysis of the TGF-β and BMP pathways (that both converge on SMAD4) revealed significantly reduced expression of TGF-β and BMP ligands in both untreated and castration resistant prostate cancer (Figure 6, Supplementary Figure 3). Other genomic alterations in *SKIL*-positive tumors included *TP53* mutation in CRPC_348, hemizygous *TP53* deletion in TCGA-KK-A8IL and TCGA-YL-A8SJ, hemizygous *PTEN* deletion in TCGA-YL-A8SJ, hemizygous *NKX3-1* deletion in TCGA-HC-7211 and TCGA-YL-A8SJ, and an *MLL3* frameshift deletion mutation in CRPC_348 (Supplementary Table 4).

**Figure 7:**
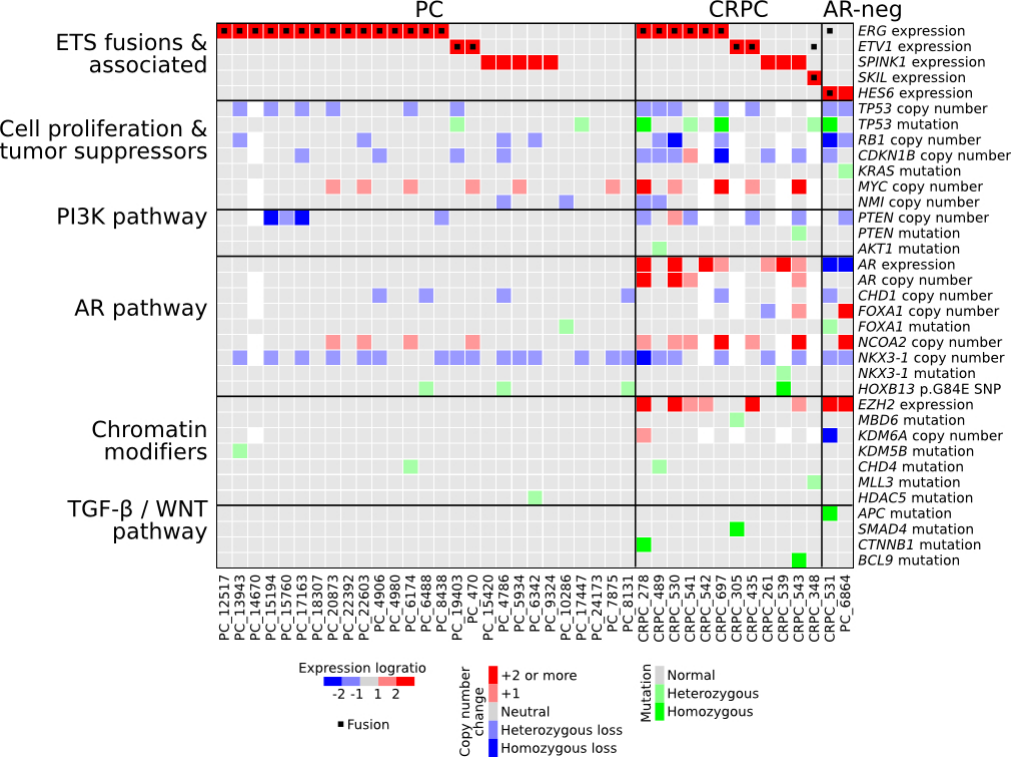
Characterization of genomic and transcriptomic changes in our prostate cancer sequencing cohort Expression and copy number changes are shown in blue and red. Point mutations and indels affecting protein coding sequences are shown in green. White squares indicate missing data.

In addition to *SKIL* rearrangements, we found various other genomic alterations in our sequencing cohort (Figure 7, Supplementary Figure 4, Supplementary Table 5). Since the original sequencing did not include paired normal tissues, we used targeted sequencing on paired blood samples to filter out germline variants. The tumor suppressor *TP53* was nonsynonymously mutated in 2 PCs and 5 CRPCs, with additional loss-of-heterozygosity in 3 CRPCs. *PTEN* was disrupted by a stopgain mutation in one CRPC sample and deleted in eight other tumors. The AR negative tumor PC_6864 carried a *KRAS* p.G12R mutation that is known to cause constitutive KRAS activation in cancers of the colon, pancreas and lungs, but is less common in prostate cancer [18]. Sample CRPC_489 harbored two distinct *AKT1* mutations, one of which (p.E17K) has been associated with dysregulated tissue growth in the Proteus syndrome [19]. We also identified somatic mutations that altered the forkhead domain and the N-terminal transactivation domain of the AR cofactor *FOXA1.* Four samples were positive for the *HOXB13* p.G84E germline variant that has been associated with prostate cancer susceptibility [20], including one homozygous sample. One AR-negative CRPC sample had acquired a DOT1L-HES6 fusion. We and others have shown that HES6 overexpression is sufficient to induce completely androgen independent growth in prostate cancer cells [21,22]. In both PC and CRPC, we identified frequent alterations in chromatin modifiers including nonsynonymous mutations in *CHD4*, *MLL3*, *HDAC5*, *KDM5B* and *MBD6,* and a homozygous deletion of *KDM6A* in one sample (Figure 7).

## DISCUSSION

SKIL (also known as SnoN) is a 684 amino acid nuclear protein that is ubiquitously expressed in human tissues [23,24] and shares its domain structure with SKI, a protein that was originally discovered through its similarity with the transforming component of the Sloan-Kettering Virus [25]. Overexpression of either *SKI* or *SKIL* in chicken embryo fibroblasts is sufficient to induce oncogenic transformation [26], and SKIL expression is elevated in many human cancers, including cancers of the skin, breast, colon and blood [27]. The 3q26 locus is amplified in several cancer types, and *SKIL* (along with *TLOC1*) has been highlighted as the most potent oncogene in this region [28]. Both SKI and SKIL contain an 80 aa SAND-like domain that can bind with the MH2 domain of SMAD4 [29]. SMAD4 (co-Smad) is an irreplaceable part of the heteromeric SMAD complexes that act as downstream mediators of TGF-β signaling. These heteromeric complexes are formed when SMAD4 binds with one or more receptor SMADs such as SMAD2 and SMAD3 [29]. The complex then translocates to the nucleus and activates transcription of TGF-β responsive genes. Binding of SKI/SKIL with the SMAD4 MH2 domain inhibits this transcriptional activation by preventing SMAD complex formation [29] or by recruitment of the nuclear co-repressor NCOR1 [16]. In addition to its binding with SMAD4, SKIL can also bind with the MH2 domains found in receptor SMADs through a domain located close to its N-terminal [16]. Neither SKI nor SKIL has been shown to directly bind DNA, despite both proteins containing a Dachshund homology domain that shares features with the forkhead/winged-helix family of DNA binding proteins [27].

In addition to the inhibitory role of SKIL on TGF-β signaling, a recent study has proposed that SKIL may play a role in regulating epithelial-to-mesenchymal transition (EMT) by inducing expression of SNAI2 (SLUG), a master regulator of EMT [28]. The same study also found that SMAD4 knockdown increased the invasiveness of human mammary epithelial cells, while SKIL overexpression had no effect on cell growth, in agreement with our findings [28]. Another recent study has proposed that SKIL can interact with and promote the activity of estrogen receptor α in the nuclei of breast carcinoma cells. The interaction occurs via two highly conserved nuclear receptor binding LxxLL-like motifs in SKIL [30]. This finding is intriguing as it suggests a potential interaction between SKIL and androgen receptor, as some LxxLL motifs can bind with the ligand binding domain of AR [31].

The SKIL rearrangements reported in this paper occurred in both untreated and castration resistant prostate cancers, and involved the 5′ partner genes *TMPRSS2*, *SLC45A3*, *MIPOL1*, *ACPP, MIPEP* and *HMGN2P46*. The androgen regulated genes *TMPRSS2* and *SLC45A3* are the most common 5′ partners involved in ETS fusions in prostate cancer [6]. *MIPOL1* is another androgen regulated gene that is involved in *MIPOL1-ETS* rearrangements in prostate cancers [32]. Expression of *ACPP* is androgen regulated and highly prostate specific among normal tissue types [33]. *MIPEP* is not generally considered androgen regulated or highly expressed in the prostate, but we observed high expression of both *MIPEP* and *SKIL* in the *MIPEP-SKIL* positive sample. *HMGN2P46* is a pseudogene that is strongly expressed in AR-expressing prostate cancers but not expressed in AR-negative cancers in our cohort, suggesting an androgen regulated promoter. We conclude that six *SKIL* rearrangements involved an androgen regulated promoter, and one involved an otherwise highly active promoter.

The discovery of *SKIL*-activating rearrangements in both untreated and castration resistant prostate cancers suggests that *SKIL* rearrangements may represent an early event in prostate tumorigenesis. This hypothesis is supported by the fact that *SKIL* rearrangements appear to be mutually exclusive with ETS fusions, which represent a known early event in prostate cancer progression [34]. The complex structure of two rearrangements highlights the fact that rearrangements can affect gene expression in a clinically significant manner without disrupting the transcribed portion of a gene. As an example of this complexity, one *SKIL*-activating rearrangement in our study juxtaposed an active promoter to a position downstream of *SKIL* in opposite orientation, and still resulted in strongly elevated expression of full length *SKIL* transcript.

Based on the cohorts studied in this manuscript, we estimate that SKIL-activating rearrangements are found in 1-2% of diagnosed prostate cancers. Due to the high incidence of prostate cancer, this fraction translates to an estimated 10,000 diagnoses and 3,000 deaths caused by *SKIL*-positive prostate cancers per year worldwide [1]. Whether *SKIL*-positive prostate cancers differ in their clinical course from other prostate cancers remains to be evaluated in a larger study. Since 6 of 7 *SKIL* rearrangements involved androgen regulated promoters, we expect that existing treatment modalities based on androgen ablation will be effective at treating *SKIL*-rearranged cancers. Nonetheless, SKIL provides an intriguing new molecular target for personalized therapy, and highlights the role of TGF-β signaling in prostate cancer progression.

## MATERIALS AND METHODS

### Sequencing cohort

Fresh-frozen tissue specimens from 12 benign prostate hyperplasias, 28 untreated prostate cancers, and 13 castration resistant prostate cancers were acquired from Tampere University Hospital (Tampere, Finland). Untreated prostate cancer samples were obtained by radical prostatectomy and locally recurrent CRPCs by transurethral resection of the prostate. Samples were snap-frozen and stored in liquid nitrogen. Histological evaluation and Gleason grading were performed by a pathologist based on hematoxylin/eosin-stained slides. All samples contained a minimum of 70% cancerous or hyperplastic cells. The use of clinical material was approved by the ethical committee of the Tampere University Hospital and the National Authority for Medicolegal Affairs. Written informed consent was obtained from the subjects.

### Validation cohort

76 additional hormonally untreated PC prostatectomy samples were acquired from the Tampere University Hospital (Tampere, Finland). Samples were snap-frozen and stored in liquid nitrogen. Histological evaluation and Gleason grading were performed by a pathologist based on hematoxylin/eosin-stained slides. Samples were confirmed to contain a minimum of 70% cancerous or hyperplastic cells by hematoxylin-eosin staining. Mean age at diagnosis was 62.1 years (range: 47.4-71.8), mean PSA at diagnosis was 11.8 (range: 3.15-51.5). The use of clinical material was approved by the ethical committee of the Tampere University Hospital and the National Authority for Medicolegal Affairs. Written informed consent was obtained from the subjects.

### Cell lines and xenografts

Prostate cancer cell lines PC-3, LNCaP, DU145, 22Rv1 and immortalized prostate epithelial cell line RWPE-1 were obtained from American Type Cell Collection (Manassas, VA, USA). LAPC-4 cell line was kindly provided by Dr. Charles Sawyers (University of California at Los Angeles, Los Angeles, CA, USA), VCaP and DuCaP by Dr. Jack Schalken (Radboud University Nijmegen Medical Center, Nijmegen, the Netherlands), and EP156T by Dr. Varda Rotter (Weizmann Institute of Science, Rehovot, Israel). All cell lines were cultured under recommended conditions. 22 previously established LuCaP-series xenografts were provided by R.L.V. These xenografts have been derived from primary and metastatic human prostate cancer and are maintained *in vivo* [35].

### DNA and RNA extraction for sequencing cohort

Fresh-frozen tissue blocks were cut into 10×20-micrometer sections using a cryotome. RNA and DNA were isolated using an AllPrep RNA/DNA minikit (Qiagen, Valencia, CA, USA) according to manufacturer's protocol. For some samples, more total RNA was isolated using Trizol (Invitrogen, Carlsbad, CA, USA) extraction according to manufacturer's protocol. Three CRPC samples had RNA extracted using both Trizol and Qiagen AllPrep. The isolated RNA was quantified by based on 260 nm absorbance and its purity assessed by the 260/280 nm ratio. Integrity was checked using Bioanalyzer (Agilent Technologies, Santa Clara, CA, USA). Reverse transcription of RNA to cDNA from clinical samples was carried out using SuperScript(TM)III (Invitrogen, Carlsbad, CA, USA) reverse transcriptase and AMV (Finnzymes, Espoo, Finland) for cell line samples with random hexamer primers according to manufacturer's instructions.

### Whole genome library construction and sequencing

Genomic DNA was sonicated into 500 bp fragments using a Covaris E210. Overhangs were converted into blunt ends using T4 DNA polymerase and Klenow enzyme. An adenine was added to the 3′ end of the blunt phosphorylated DNA fragments, and adapters were ligated on both ends. Ligated products were purified by agarose gel electrophoresis followed by QIA quick gel extraction, to remove residual free and self-ligated adaptors and to select properly sized templates for cluster generation. DNA fragments with adapters on both ends were amplified using two primers that annealed to the adapters. PCR products were checked and purified by agarose gel electrophoresis. The fragment size and molar concentration of each library was determined using Agilent 2100 Bioanalyzer and ABI Real-Time PCR System (StepOnePlus^TM^), respectively. As mean fragment size increased to 622 bp after adapter ligation, fragments between 600 bp and 684 bp were selected. An Illumina Cluster Station was used to hybridize samples onto a flow cell and amplify them for sequencing on Illumina HiSeq™ 2000. Raw image files were processed by Illumina pipeline for base-calling with default parameters resulting in 90 bp paired end reads. Reads with too many N bases (>10%) or low base quality (>50% bases with base quality <5) were discarded. Library construction and sequencing was performed at the Beijing Genomics Institute (BGI), Hong Kong.

### Whole transcriptome library construction and sequencing

Beads with Oligo(dT) were used to isolate poly(A) mRNA after collection of total RNA. Fragmentation buffer was added to shear mRNA into short fragments and to synthesize the first-strand cDNA with random hexamer primers. The second-strand cDNA was synthesized using buffer, dNTPs, RNaseH, and DNA polymerase I, respectively. Short fragments were purified with QiaQuick PCR extraction kit and resolved with EB buffer for end reparation and poly(A) addition. After that, the short fragments were ligated to sequencing adapters and suitable fragments were selected for the PCR amplification as templates and separated with agarose gel electrophoresis before sequencing. Raw image files were processed by Illumina pipeline for base calling with default parameters resulting in 90 bp paired end reads. Reads with too many N bases (>10%) or low base quality (>50% bases with base quality <5) were discarded. Library construction and sequencing was performed at the Beijing Genomics Institute (BGI), Hong Kong.

### Fusion gene analysis

To achieve robust results, fusion gene discovery was performed using two different strategies. First, we applied ChimeraScan [36] to the raw FASTQ format sequencing data. ChimeraScan used an installed instance of Bowtie 0.12.8 [37] for read alignment. Anchor length was specified as 25 bp. One nucleotide mismatch was allowed in the initial alignments and in the alignment of discordant reads. Fusion gene candidates with less than 20 spanning reads were filtered out in order to focus the analysis on strongly expressed fusion genes.

Second, we used an in-house fusion detection algorithm called Breakfast to validate the ChimeraScan results and to search for more complex rearrangement. The Breakfast algorithm operates on aligned SAM files, and therefore we first aligned our whole transcriptome sequencing reads against the GRCh37 human reference genome using Tophat version 2.0.4 [38]. Breakfast searched the alignments for discordant read pairs and unaligned individual mates. For discordant read pairs, we required the mates to be at least one megabase apart. The alignment quality of both mates in a discordant pair was required to be above 15 phred. Next, individual unaligned mates were split into two 25 bp anchors that were extracted from both ends of each 90 bp mate. The 25 bp anchors were then re-aligned against the GRCh37 human reference genome using Bowtie 0.12.8 [37], and the resulting alignments were searched for evidence of discordantly aligned anchor pairs. Breakfast then constructed clusters of evidence for chromosomal rearrangements using both discordant read pairs and anchor pairs. To produce the final list of rearrangement candidates, we filtered out any rearrangements that were not supported by at least 1 paired read and 5 anchor pairs, or by at least 20 anchor pairs.

### Sanger sequencing of TMPRSS2-SKIL fusion

The *TMPRSS2-SKIL* junction was amplified from cDNA using Phusion™ High-Fidelity DNA polymerase (Finnzymes, Espoo, Finland) and primers 5′-AGTAGGCGCGAGCTAAGCAG-3′ (forward) and 5′-CAATGCAATGGTCTGGTTTG-3′ (reverse). PCR cycling was performed as follows: 98 °C for 30-60 seconds followed by 35 cycles of 98°C for 7 seconds, 56-58°C for 30 seconds, 72°C for 30 seconds, and a final extension for 5 minutes using GC-buffer, with a final volume of 25 μl. The size of each amplicon was verified with 1% Agarose gel. Target amplicons were purified using QIAquick PCR purification columns (Qiagen Inc, Valencia, CA, USA) and then sequenced using the BigDye® Terminator v3.1 Cycle Sequencing Kit (Applied Biosystems, Foster City, CA, USA) and the ABI PRISM® 3100 sequencer (Applied Biosystems, Foster City, CA, USA) according to manufacturer's instructions.

### SKIL expression analysis using qRT-PCR

cDNA synthesis was performed from 1 μg of total RNA using SuperScript(TM) III Reverse Transcriptase (Invitrogen, Carlsbad, CA, USA) and random hexamer primers (Thermo Scientific, Waltham, MA, USA) according to manufacturers' instructions. Synthesized cDNA was diluted 1:20 to nuclease free water and expression was measured using Bio-Rad CFX96 Real Time System. The final reaction mixture (22 μl) contained 2 μl cDNA, 0.125 μl forward (5′-AGAGGCTGAATATGCAGGACA-3′) and reverse (5′-CCAAAGCAAGCAACAAACAA-3′) primers and 11 μl 2X SYBR Green qPCR Master Mix and RNAse free ddH2O. NTC (No Template Control) was used during the reaction to detect DNA contamination. PCR cycling was performed as follows: 95 °C for 20 seconds followed by 55 cycles of 95°C for 10 seconds, 58°C for 10 seconds, 72°C for 8 seconds, followed by melting curve analysis. Finally the size of the amplicon was checked using 1.5% agarose gel electrophoresis. *SKIL* expression was normalized relative to the TATA-box binding protein *(TBP*) reference gene.

### Fluorescence in situ hybridization

Frozen 5-7 μm sections of prostate cancer tissue were fixed by Carnoy fixation (Carnoy fixative: 1/3 acetic acid glacial/methanol, 50%, 75%, 2 × 100%, 15 min each), denatured in 70% formamide/2xSSC at 72-75°C for 3-5 min and air dried. BAC clones were obtained from Life Technologies. Fusion probes (RP11-814F13, upstream of *TMPRSS2*, and RP11-922G14, overlapping and downstream of *SKIL*) as well as break-apart probes (CTD-2562E3, upstream of *SKIL*, and RP11-469J4 downstream of *SKIL*) were used. The probes were labeled by nick translation with ChromaTide Alexa Fluor 594-5-dUPT or digoxigenin-11-dUTP (Roche Diagnostics, Basel, Switzerland). After hybridization for two days at 37°C the slides were washed and stained with anti digoxigenin-FITC. The slides were embedded in Vectashield antifade solution (Vector Laboratories, Burlingame, CA, USA) containing 0.1M 4,6-diamidino-2-phenylindole (DAPI) as a counterstain and the signals were scored with an Olympus BX5 epifluorescence microscope equipped with a charge-coupled device camera. Stacks of seven images were captured with each filter set with Image-Pro Plus 6.1 software (Media Cybernetics, Inc., Rockville, MD, USA) and combined to produce an RGB image with an extended depth of focus.

### SKIL western blot

Cells were lysed in RIPA buffer (Thermo Scientific) including protease and phosphatase inhibitors (Halt Protease&Phosphatase Inhibitor cocktail, Thermo Scientific). Protein concentrations were measured with DC Protein Assay (BioRad), 4x SDS sample buffer was added and 40 μg of protein lysate was loaded into each well. SDS-PAGE and protein transfer to nitrocellulose membranes were carried out according to standard protocols. Blocking was achieved with 5% milk, TBS, 0.1% Tween. Anti-SnoN (dilution 1:1000, ab128079, Abcam), anti-β-tubulin I (dilution 1:20000, T7816, Sigma-Aldrich), and polyclonal HRP-conjugated rabbit anti-mouse (dilution 1:2000, P0161, Dako) antibodies were used for protein detection.

### SKIL immunohistochemistry

SKIL-protein levels were validated from paraffin-embedded prostate cancer tissues using monoclonal mouse antibody (1:700, [2F6] (ab128079), Abcam plc, Cambridge, UK) with Power Vision+ Poly-HRP IHC kit (Immunologic, AD Duiven, the Netherlands) according to the manufacturer's instructions. Prior to staining, sections were deparaffinized and autoclaved in 10 mM of sodium citrate buffer, pH 6.0. Slides were scanned with an Aperio ScanScope XT scanner (Aperio Technologies, Inc.).

### RNA in situ hybridization

FFPE tissue sections were treated according to manufacturer's instructions using RNAscope® 2.0 HD Detection Kit - BROWN (Advanced Cell Diagnostics, Inc., Hayward, CA, USA). Briefly, slides were first deparaffinized in xylene and dehydrated in 100% ethanol. Sections were then pretreated and boiled in 50mM Tris 1mM EDTA-solution containing 0.05% Tween using Lab Vision™ PT Module (Thermo Fisher Scientific Inc., Waltham, MA, USA). Next, a target probe for SKIL mRNA (P/N 427981, Advanced Cell Diagnostics, Inc.) and signal amplifiers were hybridized using HybEZ Oven (Advanced Cell Diagnostics, Inc.). A probe for Peptidylprolyl isomerase B (PPIB, P/N 427981, Advanced Cell Diagnostics, Inc.) was used as a positive control and a probe for dihydrodipicolinate reductase (DapB, P/N 310093, Advanced Cell Diagnostics, Inc.) was used as a negative control in every assay. Signal detection was performed using DAB substrate as a chromogen. Slides were counterstained with 50% Mayer's hematoxylin (Oy FF-Chemicals Ab) and blue color was intensified with TBS-Tween. Finally, slides were dehydrated in an ethanol series and mounted. Slides were scanned with an Aperio ScanScope XT scanner (Aperio Technologies, Inc.).

### Small interfering RNA knockdown of SKIL

The knockdown of *SKIL* expression was done using small interfering RNA (AM16708, ID 107695) from Ambion (Ambion, Austin, TX, USA). The siRNA sequences (5′-to-3′) were:

1: GGCAAGUAAGUCCAUAUCATT (sense) and UGAUAUGGACUUGCCTC (antisense)

2: GGCUCACAGUAGUGGUAATT (sense) and UUACCACUACUGUGAGCCTT (antisense)

Silencer® Negative Control #1 siRNA (AM4611, Ambion, Austin, TX, USA) was used as a non-targeting control. Cells were seeded into 24-well plates (30000 cells/well) in four replicates and were transfected the following day with 50 nM *SKIL* siRNA and scrambled siRNA. INTERFERin TM (PolyPlus Transfection, Strasbourg, France) and Opti-MEM were used for cell transfection.

### Growth curve analysis

Cells were plated on 12-well plate (30 000 cells on each well) as quadruplicates and each well was scanned daily using the Surveyor Software (Objective Imaging Ltd.) with a camera (Imaging Inc., Canada) attached to the Olympus IX71 (Olympus, Tokyo, Japan) microscope and the area of the attached cells in each well was computed using ImageJ Software (Wayne Rasband, National Institutes of Health, Bethesda, MD) and divided by the mean area for day 1.

### Cell invasion assay

The effect of *SKIL* knockdown in PC-3 cells and overexpression in RWPE-1 cells on cell invasion were evaluated in BioCoat Matrigel Invasion chambers (BD Biosciences, Bedford, MA, USA) coated with a basement membrane matrix. Matrigel was rehydrated in growth medium for 2 h at 37 °C and 5% CO2. Transfected cells (10,000 PC-3 or 20,000 RWPE-1) were harvested and resuspended in 1% FBS (PC-3 cells) or Keratinocyte Serum Free Medium (RWPE-1 cells), and placed in the upper chamber of the transwell. The lower chamber contained a 750 μl medium with 10% FBS and 5 μg/ml fibronectin (PC-3 cells) or 0.05 mg/ml BPE and 5 ng/ml EGF (RWPE-1 cells). The cells were then incubated for 22 h at 37 °C in 5% CO2. Cells in the top well of the upper chamber were wiped off the top membrane with cotton swabs. The membranes were then fixed with 3.7% formaldehyde (PC-3 cells) or 100% methanol (RWPE-1 cells) and stained with 1% toluidine for 15 min at room temperature for visualization of cells. Cells that had invaded to the lower surface were photographed and counted under a microscope.

### Colony formation assay

PC-3 cells transfected with *SKIL* or scrambled siRNA were grown on 6-well plates in duplicate (5000 cells/well). Briefly, base agar containing 1% agar dissolved in Ham's F-12 mixed with 20% FBS, 2% L-glutamine and 2% penicillin-streptomycin and 1 ml mixture was transferred into the well. Then the top layer containing 0.7% agar including 10% FBS, 1% L-glutamine and 1% penicillin-streptomycin was prepared. Top agar was mixed with suspended cell line and transferred over base agar. Base and top agar were covered with normal growth medium and incubated at 37°C and 5% CO2 for 15 days. After incubation, colonies were fixed with 3.7% formaldehyde and stained with 0.1% toluidine blue. Excess dye was removed by washing with 10 mM phosphate buffer (pH 7.4).

### Transfection of SKIL into RWPE-1 cells

To study the effects of SKIL overexpression, pCI-Neo HA-hSnoN plasmid (a gift from Robert Weinberg, Addgene plasmid #10908 [17]) and universal empty, pCI-Neo backbone pUNIV-plasmid (a gift from Cynthia Czajkowski, Addgene plasmid #24705 [39]) were stably transfected into RWPE-1 cells (ATCC, cultured under the recommended conditions) with jetPEI® polymer-based DNA transfection reagent (POLYPLUS-TRANSFECTION Inc., New York, NY, USA) according to the manufacturer's instructions. Transfected clones were selected with 400 μg/ml geneticin (G418, Invitrogen Inc., Carlsbad, CA, USA) over several weeks, after which *SKIL* mRNA levels were determined using qRT-PCR and the clone showing the highest overexpression of *SKIL* mRNA was selected for further analysis. Control RWPE-1 cells were transfected with empty pCI-Neo backbone vector. All transfected cells were subsequently cultured after selection in a medium containing geneticin (200 μg/ml).

### Calculation of gene expression

RNA-seq reads were aligned against RefSeq 38 human transcript sequences using Bowtie version 2.0.0-beta6 [40]. Expression values were normalized across all samples using median-of-ratios normalization. Read counts for a given gene were divided by the total length of the gene's exons (in kilobases) to correct for gene size bias. For some genes, we observed a strong and systematic expression bias associated with the two different RNA isolation methods we used (Trizol and Qiagen AllPrep kits). To correct for this bias, we took the BPH samples from which we had extracted RNA using both Trizol and Qiagen, and calculated an expression ratio for all genes by dividing the median expression in the Trizol group by the median expression in the Qiagen group. We then used an unpaired t-test to look for genes differentially expressed between Trizol and Qiagen treated samples. Genes with a p-value less than 0.0001 were considered sensitive to the RNA isolation method, and their expression levels were corrected by dividing the expression of Trizol samples with the Trizol/Qiagen expression ratio.

### Copy number analysis

DNA-seq reads were aligned against GRCh37 using Bowtie version 2.0.0-beta6 [40]. Aligned read counts were calculated within overlapping 500 bp windows along the whole genome. Coverage logratios were calculated within each window by comparing against read count averages from four BPH controls. To normalize logratios within copy neutral regions to zero, we applied a median filter of length 50 to the logratios in each sample, rendered logratio histograms for each chromosome, and took the median of the histogram modes. This value was then subtracted from all logratios for that sample. Frequently aberrant chromosomes 8, 22, X, and Y were not included in calculating the median of modes. Coverage logratios for individual genes were calculated by taking the median logratio over all intragenic windows. If a gene's length was shorter than 20 kb, the median window was extended on both sides so as to reach a length of 20 kb. Logratios were converted into copy number changes using the formula (ploidy * 2^logratio - ploidy), where ploidy was based on the chromosome in which the gene or genomic region was located. Copy number changes were further multiplied by (1 / 0.7) to correct for the estimated 70% tumor sample purity in our samples. A gene was considered to be amplified or deleted if the corrected copy number change had an absolute value above 0.5.

### Computational identification of putative somatic mutations

We first used RNA-seq data to search for mutations in transcribed loci, and then validated putative variants using DNA-seq data. RNA-seq reads were aligned against GRCh37 using Tophat version 2.0.2 [38] and Bowtie version 2.0.0-beta6 [40]. Duplicate reads were discarded using samtools rmdup, and variants were called using an in-house pipeline. Low quality alignments (MAPQ < 10) were ignored for PC and CRPC samples, but were used for variant calling in BPH control samples. A genomic site was called heterozygous alternate in PC and CRPC if at least four reads and at least 15% of all reads at that site showed an alternate allele. In BPH samples, a genomic site was considered heterozygous if at least 2 reads and at least 5% of all reads showed an alternate allele. Any variants found in BPH samples were considered to be germline variants and filtered out. We also filtered out any variants found in the 1000 Genomes project [41], the NHLBI Exome Sequencing Project (ESP6500, unpublished, http://evs.gs.washington.edu/EVS/), or the SISU project (unpublished, http://www.sisuproject.fi/). The VCF file produced by samtools was annotated using the ANNOVAR software [42] and custom scripts. For every variant, we predicted functional impact and identified features in the genomic neighborhood. We also determined whether the variant was found in the COSMIC database [43] for cancer associated mutations.

### Illumina MiSeq targeted validation of somatic mutations

Sequencing libraries were prepared using the the Illumina TruSeq Custom Amplicon Kit following the TruSeq Custom Amplicon Library Preparation Guide. For most samples, 250 ng of DNA was used. Custom probes flanking 677 target variants were designed using the DesignStudio software (Illumina). Probes were designed so as to yield amplicons with a mean size of 250 bp. Targets were extended from one probe and ligated to the second probe. Next, during two rounds of synthesis, sample-specific indexes were incorporated, producing dsDNA molecules containing two unique indexes and flanking amplification sequences. Libraries were then generated using the following PCR program: 95°C for 180 seconds, 24 cycles of 95°C for 30 seconds, 66°C for 30 seconds, 72°C for 60 seconds, and 72°C for 5 minutes, then cooling to 10°C. Resulting libraries were cleaned up (45 μl of AMPure XP beads for each library) and normalized according to manufacturer's guidelines. Library quality was verified with an Agilent Bioanalyzer (Agilent Santa Clara, CA, USA) using a DNA 1000 chip. Finally, all libraries were pooled and diluted 1:100 before loading into a MiSeq instrument (Illumina, San Diego, CA, USA) for sequencing. The reagent kit used was the MiSeq® Reagent Kit v2 (500 cycle).

### Data access

The European Genome-phenome archive database accession number for the high throughput sequencing data reported in this paper is EGAS00001000526.

## SUPPLEMENTARY MATERIAL, FIGURES












