# Supplementary material for: Recurrent SKIL-activating rearrangements in ETS-negative prostate cancer
Source: Oncotarget. 2015 Jan 31;6(8):6235–50. doi: 10.18632/oncotarget.3359 (PMC4467434; doi:10.18632/oncotarget.3359)
Supplement: Supplementary file 1 [file oncotarget-06-6235-s001.pdf]

Recurrent SKIL-activating rearrangements in ETS-negative prostate cancer

Supplementary Material

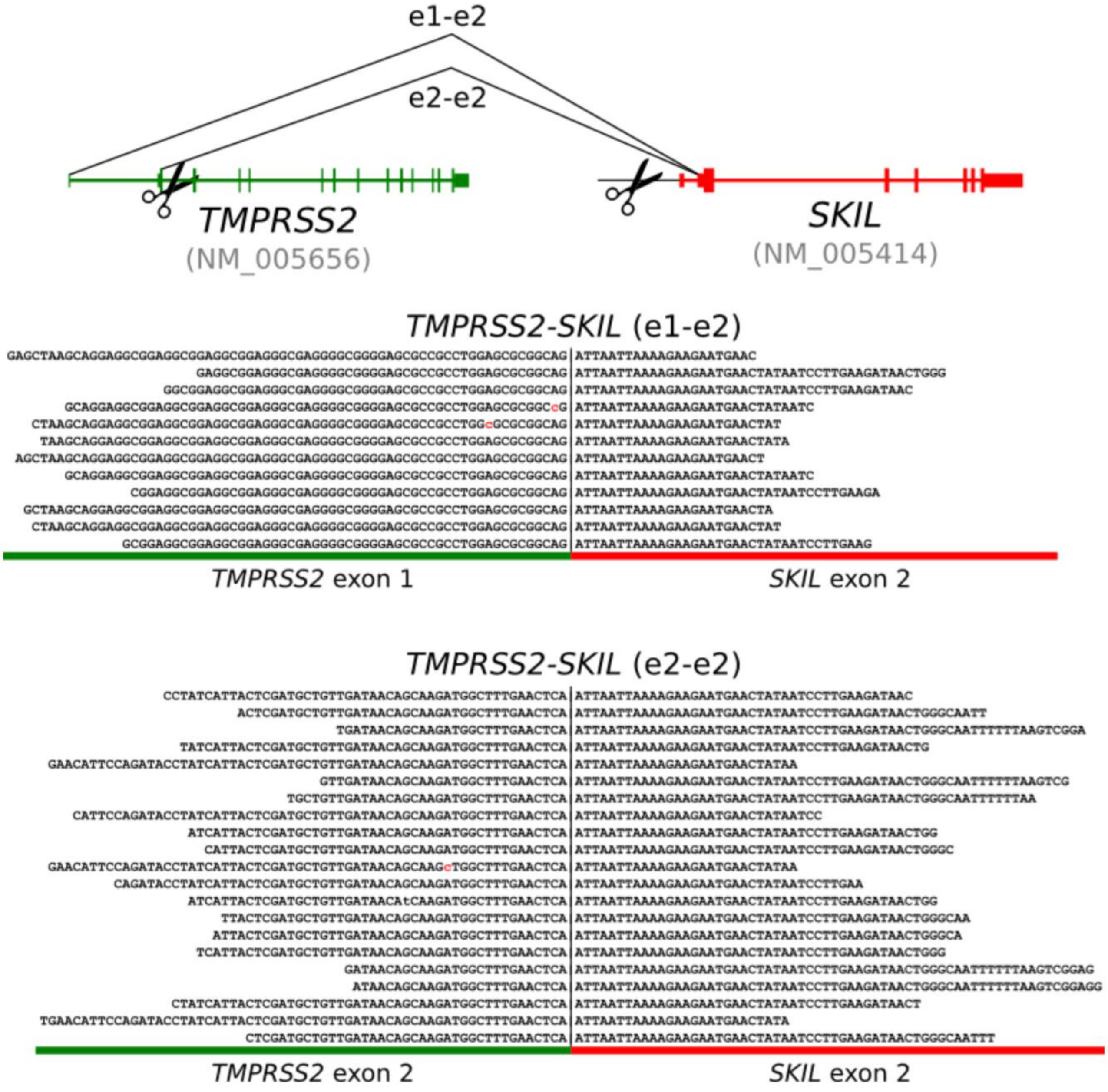

Supplementary Figure 1. Structure of *TMPRSS2-SKIL* fusion transcripts. Black lines indicate exonexon junctions with RNAseq evidence. Sequences of supporting reads are shown below. Scissor symbols indicate genomic breakpoints.

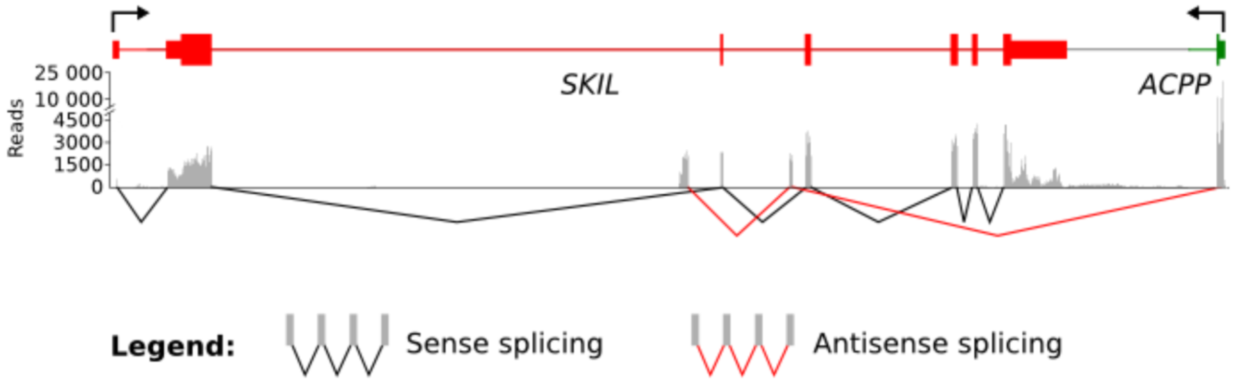

Supplementary Figure 2. Structure of the SKIL locus after *ACPP-SKIL* rearrangement. The first exon and promoter of *ACPP* is located downstream of *SKIL*, in opposing orientation. Splicing of sense and antisense transcripts is indicated with black and red lines, respectively.

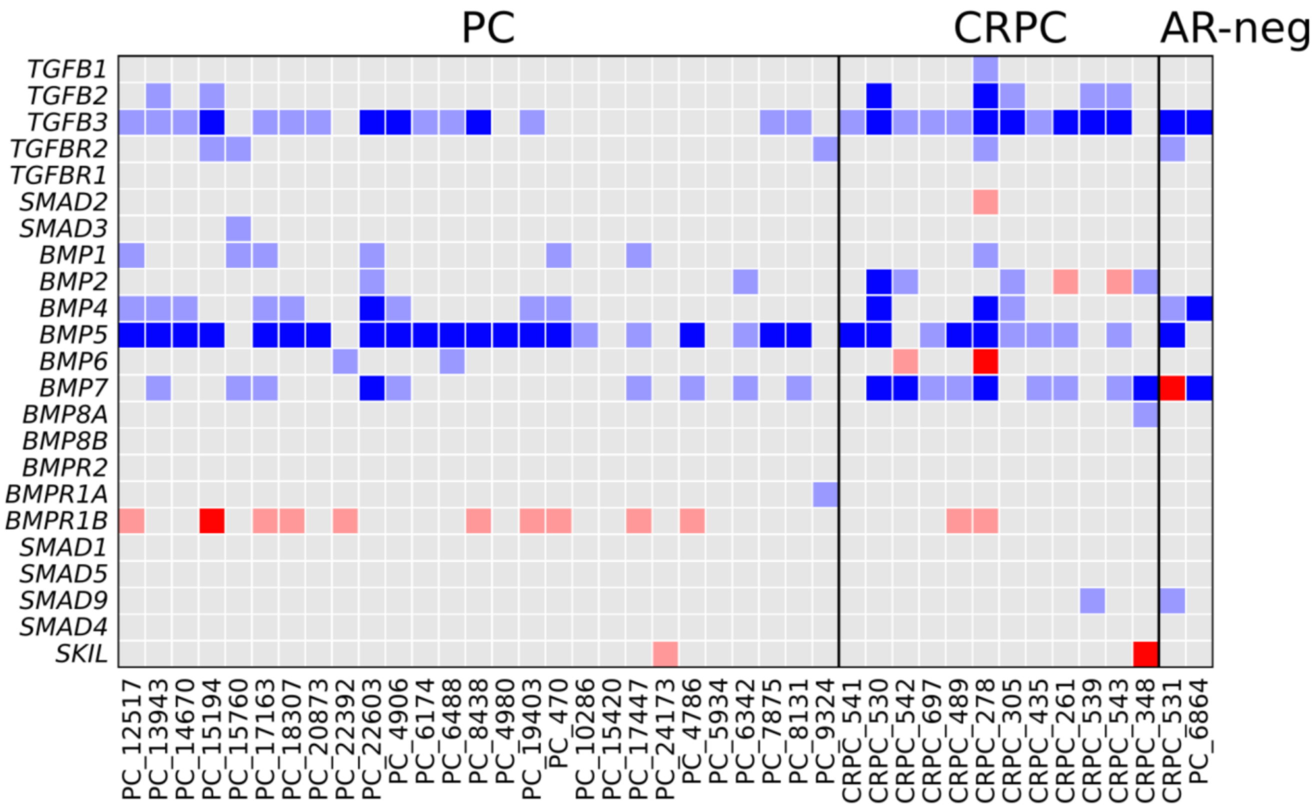

Supplementary Figure 3. Matrix showing which samples exhibited overexpression (red) or underexpression (blue) of TGF-beta and BMP pathway genes.

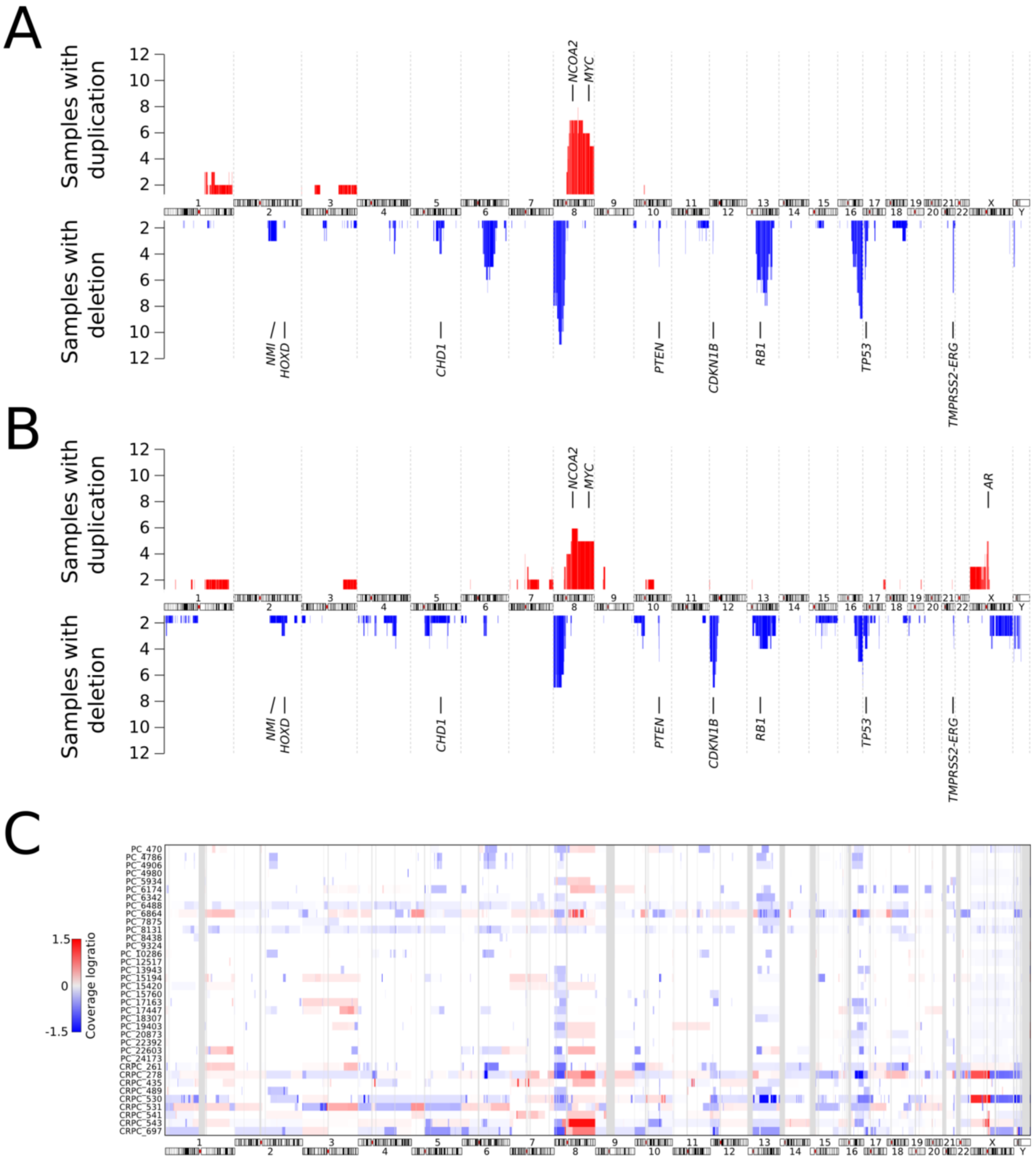

Supplementary Figure 4. Copy number landscape of untreated and castration resistant prostate cancer. (**a**) Recurrence plot showing the number of PC samples harboring a duplication (red) or deletion (blue) at a given genomic position. Positions of interesting genes are labeled in black. (**b**) Recurrence plot for CRPC samples. (**c**) Segmented copy number logratios for every sample in the sequencing cohort.
